# Characterisation of Sodium Acetate Treatment on *Acacia pennata* Natural Fibres

**DOI:** 10.3390/polym15091996

**Published:** 2023-04-23

**Authors:** Kasirajan Rajam Jaya Sheeba, Retnam Krishna Priya, Krishna Prakash Arunachalam, Siva Avudaiappan, Nelson Maureira-Carsalade, Ángel Roco-Videla

**Affiliations:** 1 PG & Research Department of Physics, Holy Cross College (Autonomous), Manonmaniam Sundaranar University, Nagercoil 627012, Tamil Nadu, India; 2 Department of Civil Engineering, College of Engineering Nagercoil, Anna University, Kanyakumari 629004, India; krishnaprakash3191@gmail.com; 3 Departamento de Ingeniería Civil, Universidad de Concepción, Concepción 4070409, Chile; 4 Centro Nacional de Excelencia para la Industria de la Madera (CENAMAD), Pontificia Universidad Católica de Chile, Av. Vicuña Mackenna 4860, Santiago 8330024, Chile; 5 Department of Physiology, Saveetha Dental College and Hospitals, Saveetha Institute of Medical and Technical Sciences (SIMATS), Chennai 600077, India; 6 Departamento de Ingeniería Civil, Universidad Católica de la Santísima Concepción, Concepción 4090541, Chile; 7 Facultad de Salud y Ciencias Sociales, Universidad de las Américas, Providencia, Santiago 7500975, Chile

**Keywords:** chemical composition, X-ray diffraction, fourier transform infrared spectroscopy, thermal stability, tensile strength

## Abstract

The present study concerns the physico-chemical, structural, mechanical and thermal characterization of Acacia pennata, a natural and almost inexpensive fibre, as a potential reinforcement in polymer composites. The effect of treating the fibre with sodium acetate to increase its qualities has been seen through the use of thermogravimetric analysis, scanning electron microscope (SEM) analysis, X-ray diffraction (XRD), mechanical property tester, and Fourier transform infrared spectroscopy (FTIR). According to XRD analysis, the elimination of lignin and wax-like impurities resulted in an increase in the AP fibre’s crystalline index (79.73%). The fibre’s thermal stability was also discovered to be 365 °C. Tensile strength (557.58 MPa) and elongation at break both increased by 2.9% after treatment with sodium acetate. The surface nature and quality of AP fibres improved after sodium acetate treatment. It was confirmed by the reduction of chemical compositions (such as hemicellulose, lignin and pectin). Given its density, the fibre can be suggested as a reinforcement in polymer composites for light-weight applications because its lightweight property will be more useful for composite manufacturing.

## 1. Introduction

In recent years, interest in the powerful use of bio-polymeric materials has expanded significantly. Natural fibres, sometimes referred to as non-wood lignocellulose materials, are being researched as potential environmentally friendly composites that could reduce or replace synthetic fibres and polymers. Environmentally friendly materials have come into consideration as a result of rising environmental awareness and public interest, new environmental rules, and unsustainable petroleum consumption. Natural fibre is regarded as one of the eco-friendlier materials with superior qualities to synthetic fibre [1]. Natural fibres are fibres that are not artificial or synthetic. They may come from either plants or animals [2]. The production of composite materials using natural fibres such as jute, flax, sisal and oil palm, both renewable and non-renewable resources, has attracted a lot of attention in recent decades [3,4,5].

The plants that produce cellulose fibres can be divided into a variety of different types, including bast fibres, seed fibres, leaf fibres, grass and reed fibres, and core fibres [6]. Everyone should adopt the natural fibres due to their advantage over synthetic fibres. Natural fibre cell walls, which are mostly made of chemical constituents such as cellulose, give them their strength and stiffness [7,8,9,10]. Technically, natural fibre-based composites can make use of a wide variety of fibre types from various plant species and bodily positions (such as leaves, stems, and seeds).

According to current indications, the natural fibre-reinforced polymer composites (NFPCs) global market will continue to see rapid growth. Over the past few years, the use of NFPCs in expanding industry sectors for consumer goods has increased significantly. Natural fibre-reinforced polymer matrix has drawn a lot of attention in a number of applications due to its superior advantages over synthetic fibres in terms of its good flexural modulus and tensile modulus, enhanced finishing surface of moulded parts in composites, abundance, use of renewable resources, biodegradability and flexibility during processing [11]. NFPCs with strength and higher stiffness and can be made by mixing the durable and lightweight natural fibre with polymer (thermoplastic or thermoset) [12]. On the other hand, there are glaring shortcomings and problems with natural fibres. The hemicelluloses, cellulose, pectin, lignin and waxy components that make up natural fibres allow moisture from the environment to be absorbed, resulting in flimsy bonds between the fibres. Furthermore, couplings between natural fibres and polymer are thought to be challenging because the chemical structures of the fibres and the matrix differ. These factors contribute to inadequate stress transfer at the manufactured contact of composites. Because of this, natural fibres unquestionably need special treatments such as sodium acetate, sodium hydroxide, permanganate, silane, etc. [4,5]. In the present study, we elected to treat the AP fibre with sodium acetate. These modifications frequently centre on the application of functional reagent groups (such as sodium hydroxide and ammonium sulphide) [13,14] that have the potential to interact with fibre structures and change their composition. The incompatibility between the fibre and polymer matrix is significantly improved in natural fibres because they absorb less moisture as a result of fibre changes [15]. Applications for NFPC are rapidly diversifying across many engineering specialties. Natural fibre-reinforced polymer composites have gained significant importance in a variety of automotive and civil applications [16,17]. Natural fibre composites are used in industries other than the automotive, such as in building and construction [18,19], sports, and aerospace. Panels, window frames, decking, and bicycle frames are some examples [20]. The current study aims to evaluate the extraction and physicochemical, structural, mechanical, and thermal properties of natural fibres from Acacia pennata.

## 2. Materials and Methods

Sodium acetate pellets, distilled water and fibre material. Analytical-grade sodium acetate pellets were purchased from Premier chemicals, Kanniyakumari district, Tamilnadu, India.

### 2.1. Fibre Extraction

**Figure 1:**
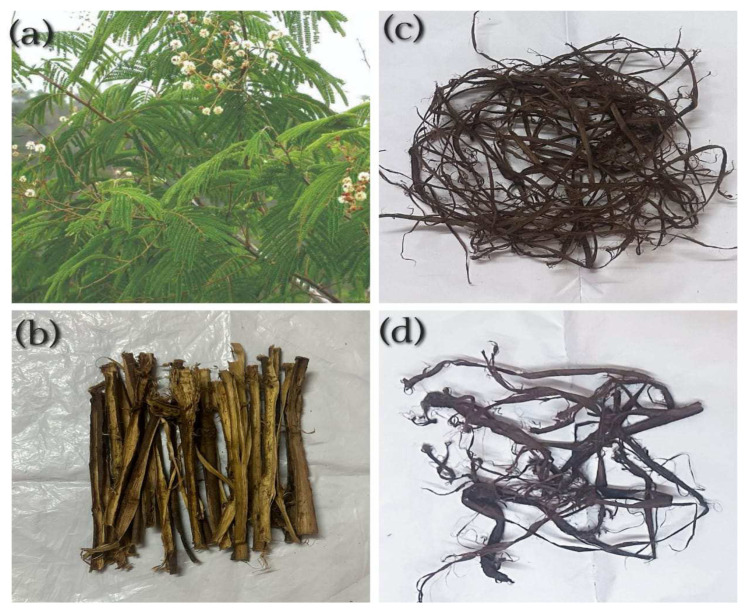
Photographs of (**a**) Acacia pennata (AP) plant, (**b**) AP stem fibre, (**c**) untreated AP fibres, (**d**) sodium acetate-treated AP fibres.

Initially, Acacia pennata (AP) fibre should be separated from the bark of the plant by following the commonly used step-by-step procedure described below [21,22]. Figure 1 shows images of the Acacia pennata plant, the stem part and the fibre treatments.

1. The required amount (around 50 to 70 grams) of fibre (for example, the bark) has to be collected and separated from the AP plant.
2. To obtain microbial degradation, the collected AP plant parts must be submerged in distilled water for about a week.
3. The fibre is then dried at room temperature to remove any remaining moisture.
4. The fibre is then taken for chemical processing. For about 20 minutes, the fibre is submerged in 0.1 M sodium acetate solution.
5. The unwanted particles on the fibre surface are then removed by washing the fibre in distilled water.
6. The fibre is then separated from the bark using a metallic comb.

### 2.2. Powder X-ray Diffraction 

An X-ray diffractometer (Bruker model D8 Advance A25) is used to gather an X ray diffraction pattern from the powdered AP fibre samples created by cutting the AP fibres into powder-like, thin strips. Utilizing CuKα radiation, a scanning speed of two per minute, and an operating temperature range of 3° to 70° Celsius, the XRD study was carried out. The crystalline index of the sample was calculated using the formula [23]

CrI (%) = [(I002 − Iam)/I002] × 100

where I002 is the maximum intensity of the peak which is observed from the maximum intensity of the crystallographic plane (002). Scherrer’s formula was used to calculate the size of the crystallite (CS).

CS = 0.9λ/(βcosθ)

where λ is the wavelength of the X-ray (1.54 Å), β is full width at half maximum (FWHM) and θ is the angle of diffraction. 

### 2.3. FTIR (Fourier Transform Infrared Spectroscopy)

Acacia pennata fibres were treated with sodium acetate and subjected to FTIR spectroscopy for analysis using a spectrophotometer (Perkin Elmer spectrum two) with a resolution speed of 32 cm^−1^ and a scanning range between 4000 cm^−1^ and 500 cm^−1^ [24].

### 2.4. Scanning Electron Microscopy (SEM)

A scanning electron microscope model Joel 6390 LV was used. Prior to SEM observations, all specimens were double-sided with an electrically conductive carbon-adhesive tab mounted on aluminium holders and sputtered with a 10 nm layer of gold.

### 2.5. Elemental Analysis

Using a CHNS analyser, the powdered jute fibres were subjected to an elemental analysis (Elementar Vario EL). Three analyses of each sample were performed.

### 2.6. Physico-Chemical Analysis

Physical analysis is typically used to find the density of natural fibres. Diameter is typically measured using a microscope, while density is typically determined using a gas pycnometer setup. A chemical analysis study was used to analyse the presence of hemicelluloses, cellulose, pectin, lignin wax, and ash content in the Acacia pennata fibre. This study is most useful for analysing the presence of the aforementioned compositions, which determine the strength and bonding capacity of the fibre materials [25,26,27]. All tests were performed in accordance with AATCC, ASTM, ISO, IS, BS and other international standards.

### 2.7. Thermal Analysis

A Perkin Elmer Diamond TG/DTA analyser (HITACHI STA-7300) was used to determine the thermogravimetry of the powdered AP samples. The 10 mg samples were placed in an alumina crucible with a pinhole at flow rates of 100 ml/min and constant heating rates of 10 °C/min to maximum temperature of around 750 °C.

### 2.8. Mechanical Analysis

The tensile testing machine (SWICK/ROELL) was used to measure the uniaxial tensile strength of the fibre material. The load applied in tensile strength analysis is 0.1 N with a crosshead speed of 30 mm per minute.

## 3. Results and Discussion

### 3.1. Powder X-ray Diffraction Analysis

**Figure 2:**
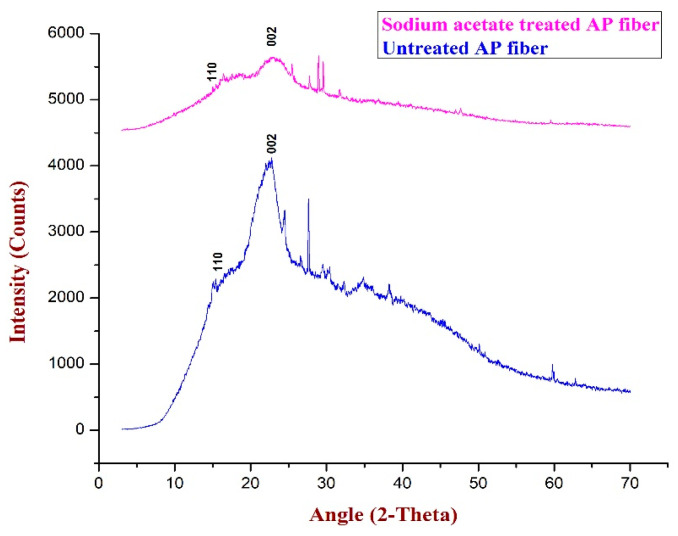
PXRD patterns of untreated and sodium acetate-treated AP fibre.

To forecast the crystallinity index value of Acacia pennata natural fibre, XRD analysis is carried out. The degree of the fibre’s crystalline nature is indicated by the crystalline index (CI). The diffraction patterns of water-treated and sodium acetate-treated AP fibres are displayed in Figure 2.

Figure 2 displays the sodium acetate-treated fibre’s wide-angle X-ray diffraction patterns. The sodium acetate-treated APFs exhibit the two major peaks at 2θ = 15.37° and 22.73° in the diffractograms. These stand for the fibre’s amorphous (110) and crystalline (002) components, respectively. It was apparent that the peak (22.73°) indicates that the sodium acetate solution entered the fibre and eliminated lignin, wax, and hemicelluloses from the fibre’s surface. The broadening peak observed around 28° could be attributed to the broadening caused by the crystallite size. The sodium acetate-treated AP fibre has a crystallinity index (CI) value of (79.73%). It was slightly higher than Althaea Officinalis fibre (68%) and slightly lower than hemp fibre (87.9%), flax fibre (70%) and sisal fibre (75%) [28,29,30]. In comparison to AP fibres treated with water (46.52%), sodium acetate-treated AP fibres had a higher crystalline index. The removal of amorphous components from the fibre is thought to have an improved crystalline nature and stress relaxation of the cellulose chains [31,32,33]. The sodium acetate-treated APFs had a crystallite size (CS) of 2.4 nm. The atomic molecules present in the sodium acetate solution have occupied the vacuum gaps of the untreated AP fibres following the sodium acetate treatment. As a result, the AP fibre’s crystallite size was increased. Table 1 lists the calculated values for the sodium acetate-treated and untreated AP fibres’ crystalline index and crystallite size.

### 3.2. FTIR Analysis

FTIR analysis is used to identify the functional groups present in the natural fibres. The peaks guarantee the presence of the O-H group consecutively moisture contents, C-H group in turn cellulose and hemicellulose contents, C=C group in turn wax contents, and C-OH group in turn lignin contents [34,35,36].

**Figure 3:**
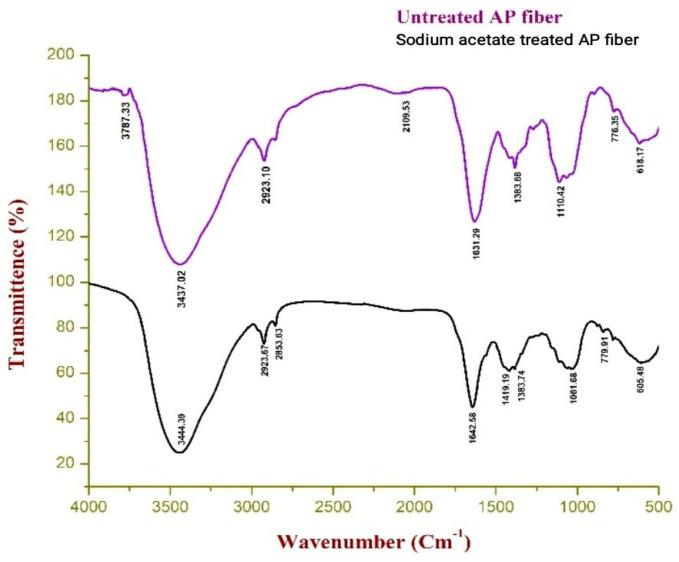
FTIR curves of untreated and sodium acetate-treated AP fibre.

According to Figure 3, the OH stretching in hydrogen bond for the broad and powerful absorbance of the region 3444.39 cm^−1^. The appearance of very strong and widespread absorption was a blatant sign that the fibre contained numerous hydroxyl groups [37]. The elongation of the C-H bond in cellulose and hemicellulose is vibrating at a wavelength of 2923.67 cm^−1^, which is the location of the absorbance band. The C-H bending due to cellulose also corresponds to a peak of APFs at around 2853 cm^−1^. The medium absorption peak at 1642.58 cm^−1^ represents the C-O stretching of lignin, and the cellulose C-H2 stretching is noted by the characteristic peak 1419.19 cm^−1^. The observed absorption peak around 1383.74 cm^−1^ supports the C-H bending. The C-O-H stretching is what causes the absorbance band seen at 1061.68 cm^−1^. Last but not least, the peaks around 605.48 cm^−1^ and 779.91 cm^−1^, which are attributed to the presence of saline content, occur outside of the plane of OH bending. The vibrational band assignments for untreated and sodium acetate-treated AP fibre are shown in Table 2 below.

### 3.3. SEM Analysis

**Figure 4:**
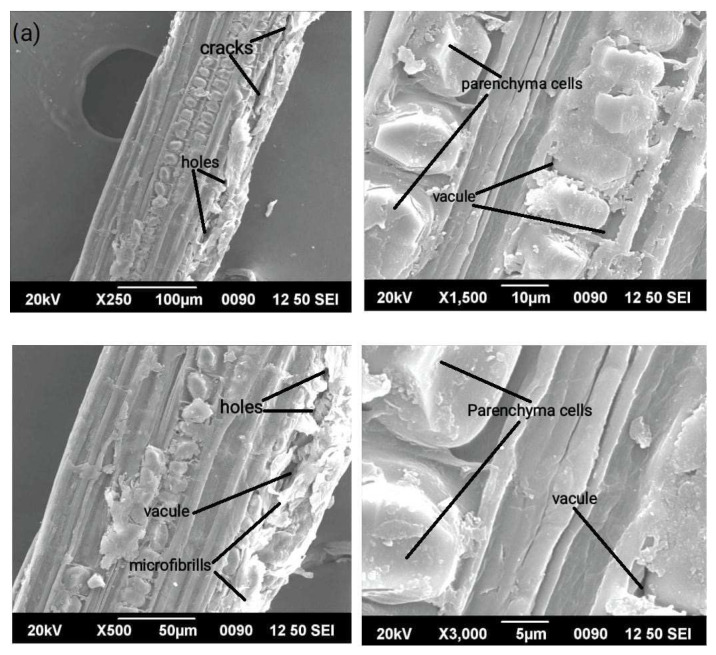

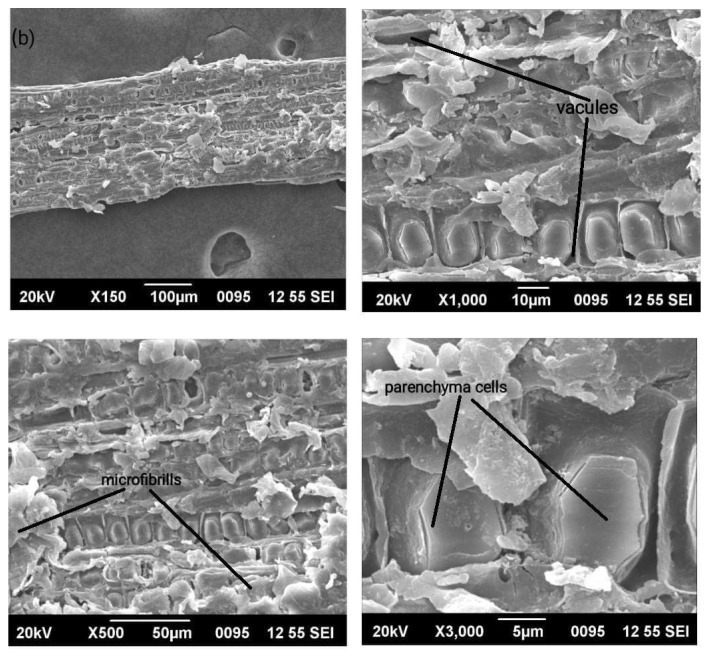
(**a**). Surface morphology of untreated AP fibre under 100, 500, 1500 and 3000 magnification fields. (**b**). Surface morphology of sodium acetate-treated AP fibre under 100, 500, 1000 and 3000 magnification fields.

The fundamental characteristics of the fibre samples are illuminated with fine clarity by the excellent results of scanning electron microscopy in identifying morphological features. Figure 4a,b detail the surface morphologies of untreated and sodium acetate-treated *Acacia pennata* (AP) fibres. Surface morphology is a crucial component that determines whether or not the desired fibre material can function well as a reinforcement material. The failure approach is frequently questioned using SEM analyses at the micro level [38]. Figure 4a,b respectively, show the morphological images of sodium acetate-treated fibres at various magnifications. These micrographs make it clear that the fibre is made of uniaxial fibres that are rough-surfaced. The magnification also makes the vacuoles of the fibre visible. The sodium acetate treatment results in a reduction of the hemicellulose content. Additionally, at higher magnification, the common parenchyma cells are clearly visible in the sodium-acetated fibres. The fibre matrix interface’s spacious bonding would be significantly improved by the removal of non-cellulosic structures, particularly wax, which create a smooth surface. However, there is still little variation, which led to the conclusion that additional treatment is required to improve the fibre material [39,40]. Although the AP fibre’s sodium acetate treatment reduced its surface roughness, it improves its morphological properties. One can determine the bonding between the fibre under study and the matrix medium by looking at the surface texture, which can be either rough or smooth. Chemical treatments will aid in roughening the fibre surface, increasing the fibre’s bonding capacity.

### 3.4. Energy Dispersive X-ray Spectroscopic Analysis (EDX)

EDX is used to measure the amount of compounds present on the surface of the fibre (such as Carbon, Oxygen, Nitrogen, etc.) [41,42,43].

**Figure 5:**
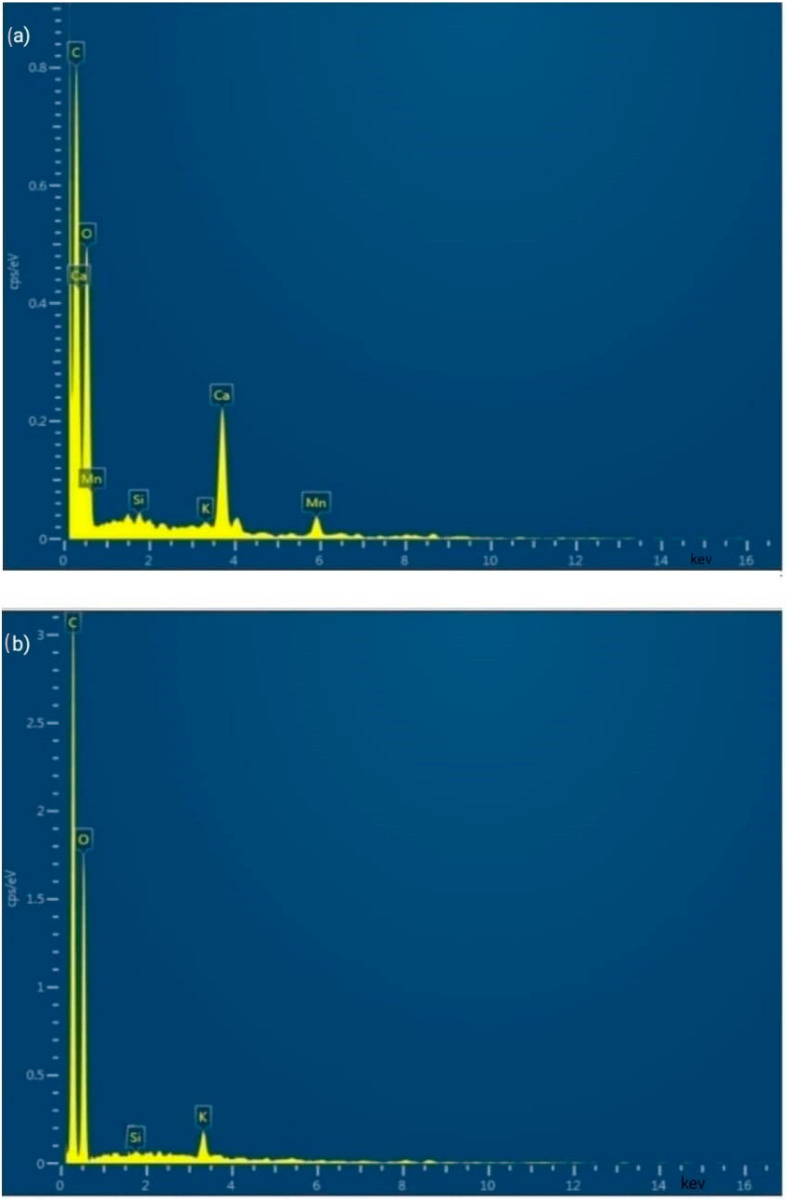
EDX pattern of (**a**)sodium acetate-treated and (**b**) untreated AP fibre.

The distribution of elements, expressed in atomic percentage and weight percentage, on the surface of AP fibres treated with sodium acetate was shown in Figure 5. In particular, the major peaks C and O on AP fibres indicate its profusion (i.e., an extremely large amount of carbon and oxygen). Additionally, this is what to expect from plant fibre. The sodium acetate treatment revealed that the carbon content had slightly increased in both weight percentage and atomic percentage. These variations might exist as a result of the treated fibre being successfully removed, as the SEM analysis also supported. A potential change in the Si peak suggests that wax-like impurities have been removed from the surface of the fibre material treated with sodium acetate. Table 3 displays the weight and atomic percentages of various compounds present in untreated and sodium acetate-treated AP fibre.

### 3.5. Thermogravimetric Analysis

**Figure 6:**
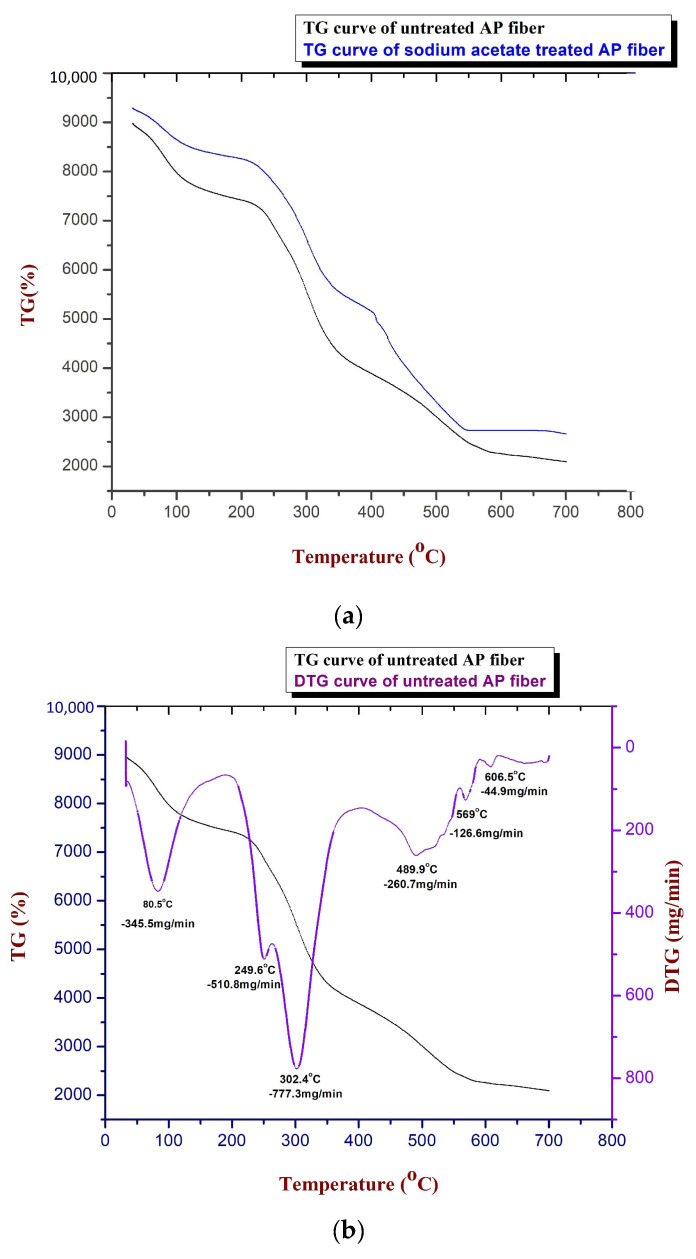

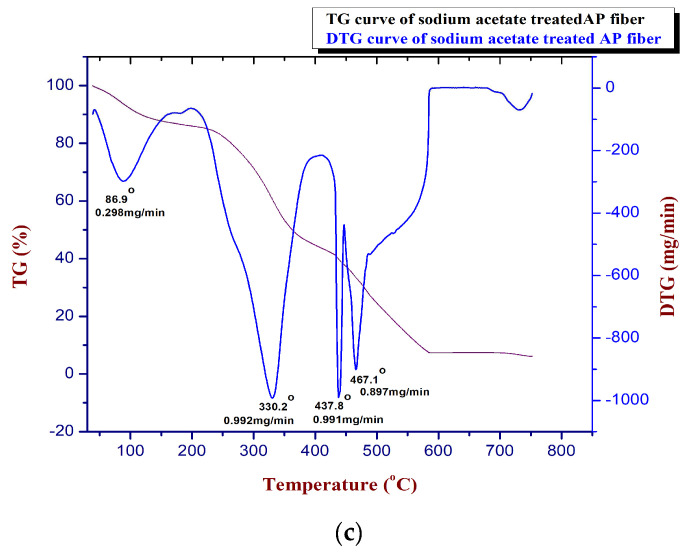
(**a**). TG curves of untreated and sodium acetate-treated AP fibre. (**b**). TG and DTG curves of untreated AP fibre. (**c**). TG and DTG graphs of sodium acetate-treated AP fibre.

Fibres are subjected to thermogravimetric analysis to determine the thermal stability and maximum degradation temperatures. Figure 6a–c depict the TG and DTG curves of untreated and sodium acetate-treated *Acacia pennata* (AP) natural fibre. 

From Figure 6a, the case of sodium acetate AP fibre, as the temperature was increased from ambient 40 °C to 120 °C, 10.12% of its weight got reduced, which corresponds to the moisture content inside the sample. This shows the first decomposition of the sample. The next degradation was observed at temperatures of around 120 °C to 280 °C and was caused by the thermal breakdown of hemicelluloses and the glycosidic bonds in celluloses. In this stage, weight loss is close to 13.59%. The complete decomposition of cellulose and hemicellulose components may be responsible for the weight loss of 32.08% that is seen between 280 °C and 400 °C. The weight has decreased here by about 32.08%. A further decrease was seen between 400 °C and 500 °C, which may be related to the lignin’s deterioration and in which 20% of the weight loss occurred. At 500 °C to 600 °C, the AP fibre’s final decomposition took place, resulting in a weight reduction of roughly 13%. A temperature of 226 °C was discovered to be the highest thermal degradation temperature. Table 4 shows the thermal study of untreated and sodium acetate-treated AP fibres.

According to DTG analysis, the rate of decomposition has been lowered to 0.298 mg/min at 86.9 °C. The next stage of degradation was noticed at 330.2 °C as a result of the fibre sample’s reduced cellulose content (nearly 0.992 mg/min). At 437.8 °C and 467.1 °C, respectively, final weight losses of 0.991 mg/min and 0.897 mg/min were noted, which were attributed to the degradation of wax and other impurities. 

### 3.6. Differential Scanning Calorimetry (DSC)

When a composite material is heated, the heat released or absorbed under chemical reactions among its constituent parts is calculated using DSC analysis. At various temperatures, a series of exothermic and endothermic reactions occur during the decomposition process [44]. The size and positioning of the exothermic and endothermic peaks reveal the thermal phase transition of *Acacia pennata* natural fibres. An exothermic event causes heat to be released, whereas an endothermic event causes the sample to absorb heat. 

**Figure 7:**
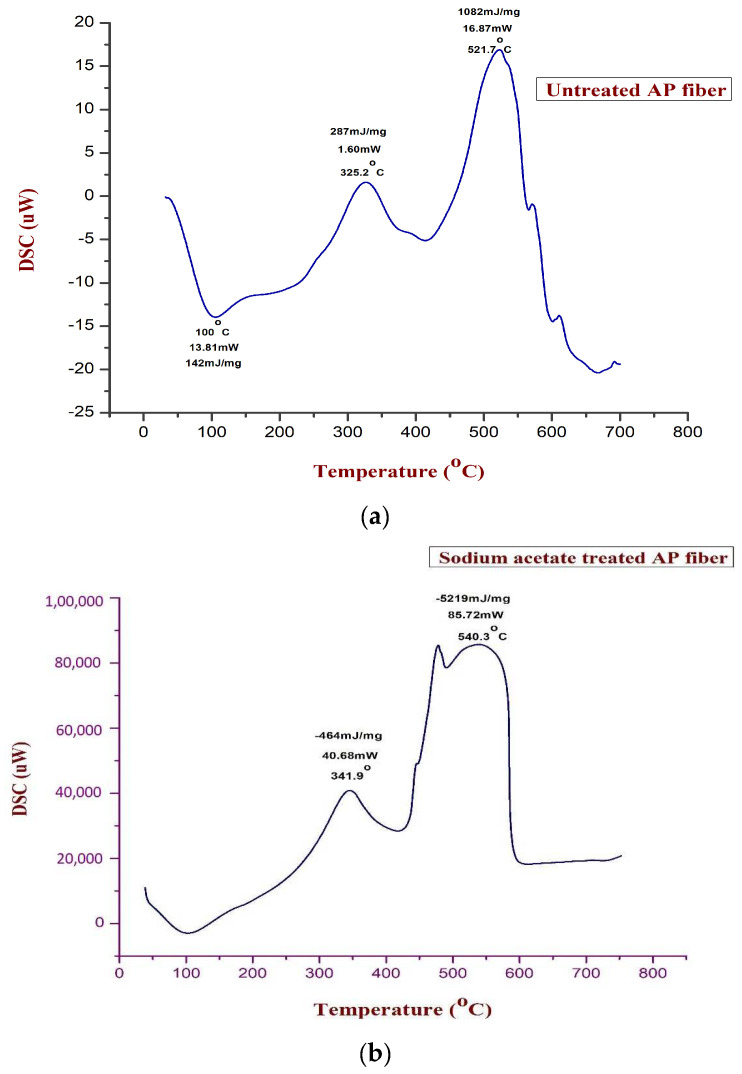
(**a**). DSC curve of untreated AP fibre. (**b**). DSC curve of sodium acetate AP fibre.

In untreated AP fibres, there are two exothermic peaks and one endothermic peak, while in sodium acetate-treated AP fibres, there are only two exothermic peaks in two different locations (see Figure 7a,b). Furthermore, the burning of hemicellulose and other components present in *Acacia pennata* natural fibres can be seen in the exothermic peak at 341.9 °C. However, in this instance, 464 mJ/mg of heat was released at a usage of roughly 40.68 mW. The removal of lignin and hemicellulose by sodium acetate treatment is responsible for the next exothermic peak, which was seen at 540.3 °C. At this temperature, 5219 mJ/mg of heat from the AP fibre sample was released using approximately 85.72 mW of energy.

Untreated fibres in this instance displayed two exothermic peaks and one endothermic peak. Lignin, hemicellulose and -cellulose decomposition reached an exothermic peak at 325.2 °C. This process used 1.60 mW of power, and 287 mJ/mg of heat were released. Another exothermic peak occurred at 521.7 °C, releasing 1082 mJ/mg of heat and requiring 16.87 mW of power to break down wax-like substances. The dehydration in the AP fibre sample corresponds to the endothermic peak at 100 °C, and the heat absorbed during this event was 142 mJ/mg. With only one exothermic peak at 469.7 °C and an alkali-treated AP fibre, 56.60 mW of power was applied for decomposition, and a 3589 mJ/mg loss was also observed. This was most likely caused by the sodium acetate treatment’s effect on the partial removal of cellulose, hemicelluloses, and lignin from the fibre; more heat was needed to remove these constituents.

### 3.7. Elemental Analysis of Carbon, Hydrogen, Nitrogen and Sulphur (CHNS) 

CHNS analysis is a type of elemental analysis used to determine the amounts of carbon, hydrogen, nitrogen, and sulphur present in a sample. This analysis involves combustion of the sample in an oxygen-rich environment, which converts all of the carbon and hydrogen to carbon dioxide and water, respectively. The nitrogen and sulphur are converted to nitrogen oxides and sulphur dioxide, respectively. The resulting gases are then separated and analysed using various techniques, such as gas chromatography, to determine the amounts of each element present. The sample is combusted to perform the CHNS elemental analysis, which is done with the help of a delicate instrument. The elemental analyser produces uniform gases of the compounds C, H, N, and S while it is burning. Testing was done using a small amount of the sample in order to analyse the purity and composition of the chemical samples. CHNS research interpreted that the treated fibre’s sulphur content was slightly reduced but the hydrogen content was completely eliminated, possibly as a result of chemical reactions on the treated fibre. The weight percentages of the various compounds in the untreated and sodium acetate-treated AP fibre samples are displayed in Table 5.

### 3.8. Chemical Analysis

To determine the quantity of chemical constituents such as cellulose, hemicellulose, lignin, pectin, moisture, wax, and ash presented in the fibre, a chemical analysis was done. This study is applicable in characterization, because the presence of the aforementioned compositions determines the strength of the fibre materials [45,46,47]. In order to achieve better mechanical strength, the cellulose content should be high in percentage, hemicellulose content should be low, lignin content should be negligible, and moisture, ash, and wax content should be low. Table 6 provides a summary of the chemical analysis performed on AP fibres treated with sodium acetate. The collected information showed that the untreated fibres have the following compositions: cellulose (52.59%), hemicellulose (14.51%), lignin (19.4%), pectin (2.92%), wax (0.93%), moisture (12.7%), and ash (5.89%). It was found that after the sodium acetate treatment, the cellulose content increased from 45.68% to 52.59% and the hemicellulose content decreased from 41.13% to 14.51. This is also evident from the PXRD analysis, which shows that the crystallinity index of AP fibres increased in sodium acetate treatment. Additionally, the AP fibre’s density dropped from 1090 kg/m^3^ to 590 kg/m^3^.

### 3.9. Mechanical Analysis

The stress at which a force causes a material to lengthen before breaking is known as tensile strength. The breaking strength in tension for an axially loaded material is given by s = P/a, where P is the breaking force, ‘a’ is the cross-sectional area, and ‘s’ is the breaking strength. Pounds per square inch, or psi, is a common unit of measurement for tensile strength [48]. The experimental fibres’ breaking strength, breaking elongation, and tensile strength are shown in Table 7. When the APFs are treated with sodium acetate, calculations show that the tensile properties are favourable. Comparing chemically treated APF to water-treated APF, the tensile strength of the chemically treated APF rises by 557.58 MPa (181.694 MPa). This demonstrates that treating AP fibres with sodium acetate increases their tensile strength. Additionally, these treatments resulted in a decrease of Young’s modulus (19.23 GPa) and microfibrillar angle (12.17°) but the elongation at break (2.9%) also fell. These findings showed that all of the AP fibres treated with sodium acetate underwent chemical changes that caused variations in their strength. Due to chemical treatment, which is also seen in this (AP) cellulosic fibre, the tensile strength and modulus increased [49]. From the literature review, the tensile strength and Young’s modulus of sodium acetated-treated flax and kenaf fibre composites are (73.7 MPa and 6.94 GPa) and (55.8 MPa and 4.7 GPa) [50,51] which shows an increase in mechanical strength similar to our experimental results. The quality of *Acacia pennata* (AP) fibres was significantly improved by using a sodium acetate solution for their treatment. This treatment effectively removed impurities, including 80% of the hemicellulose content, holes, and other impurities, resulting in a smoother and better surface quality of the fibres. The treatment also resulted in an increase in hydrogen bonding between the cellulose chains of the AP fibres, which contributed to their good mechanical performance, as seen in the FTIR spectrum. This increased hydrogen bonding was a result of the reaction of the hydroxyl groups on the fibre surface with the acetate and sodium ions in the solution. The resulting removal of impurities and improved hydrogen bonding led to an improvement in mechanical properties, such as tensile strength and modulus. In addition to improving mechanical properties, the sodium acetate treatment also enhanced the surface smoothness of the AP fibres by reducing impurities and promoting a reaction with the fibre surface. Overall, the sodium acetate treatment improves the quality of AP fibres by removing impurities, increasing hydrogen bonding, and improving mechanical properties and surface smoothness. It is important to note that further investigations should be conducted to fully understand the impact of this treatment on the AP fibres. In the future, these sodium acetate-treated AP fibres may be used to create composites for prospective uses.

## 4. Conclusions

In this study, the physico-chemical, structural, mechanical, and thermal characteristics of the new cellulosic fibres obtained from the *Acacia pennata* plant (AP) were investigated. The fibres were subjected to water and 0.1 M sodium acetate treatment, and their physical properties, diameter, density, and various characterization studies were conducted. The findings revealed that the AP fibre is a lightweight material with a density of 0.59 kg/m^3^, making it suitable for composite production. 

The PXRD analysis indicated that the crystallinity of the fibre was improved with the removal of impurities during the treatment process, and the sodium acetate-treated fibre exhibited a better crystallinity index, which is beneficial for composites with potential applications. The FTIR analysis showed that the fibre contained hydrogen molecules and revealed the vibrations around the wave numbers 3600–3000 cm^−1^ due to O-H stretching. 

CHNS analysis was conducted using an elemental analyser to determine the weight percentages of nitrogen, carbon, and hydrogen. The SEM-EDAX analysis showed that the surface roughness of the AP fibres improved after sodium acetate treatment, and the non-cellulosic components disappeared from the surface, giving an ordered parallel arrangement. The TG-DTA analysis revealed the thermal behaviour of the fibre at varying temperatures, and the sodium acetate treatment increased the mechanical strength of the AP fibre, as evidenced by the tensile analysis.

In conclusion, the sodium acetate treatment of the fibres improved their characteristics, making them suitable as raw materials for composite reinforcement. The investigation on KMnO_4_-treated *Acacia pennata* fibres also yielded impressive mechanical properties, including a high tensile strength and Young’s modulus, indicating their potential use in construction, heat-shielding components, and interior structural components. However, further research is required to assess their feasibility and performance in comparison to existing materials in the construction and composite industries.

## Figures and Tables

**Table 1:** CI and CS of untreated and sodium acetate-treated AP fibre.

Sample	Crystallinity Index (CI) (%)	Crystallite Size (CS) (nm)	Standard Deviation
Untreated AP fibre	46.52	1.9	9.51
Sodium acetate-treated AP fibre	79.73	2.4	2.88

**Table 2:** FTIR vibrational band assignments of untreated and sodium acetate-treated AP fibre.

Wave Number (cm^−1^)		Vibrational Band Assignments
Untreated	Sodium Acetate-Treated	
3914.58, 3787.33, 3437.02	3444.39	-OH Stretching in Hydrogen bond
2923.10	2923.67	-CH Stretching of Cellulose
-	2853.63	-CH Stretching of cellulose and hemicelluloses
2109.53, 1631.28	-	-C=C Stretching
-	1642.58	-CO stretching
-	1419.19	-CH_2_ stretching of cellulose
1383.68	1383.74	-CH bending
1271.83	-	-CO stretching in lignin
1110.42	-	-C=O=C pyranose ring skeletal vibration of cellulose
-	1061.68	-COH stretching of lignin
-	1033.85	Symmetric CO stretching of lignin
776.35	779.91	Presence of saline content
618.17	605.48	Out of plane of -OH bonding

**Table 3:** Weight % and atomic % of compounds in untreated and sodium acetate-treated AP fibre.

	Untreated		Sodium Acetate-Treated	
Element	Wt %	Atomic %	Wt %	Atomic %
C	55.03	60.03	55.22	60.61
O	42.93	38.02	41.62	36.98
Si	0.29	0.14	0.32	0.16
K	1.74	1.21	0.14	0.05
Ca	-	-	1.81	1.71
Mn	-	-	0.88	0.49
Total	100	100	100	100

**Table 4:** Thermal study of untreated and alkali-treated AP fibres.

Type of Fibre	Temperature during Loss (°C)	Weight Loss (%)	Standard Deviation (min)	Residual Char at 750 °C
Untreated AP fibres	40–120	13.5	0.24	0.024
	120–280	14.5		
	280–400	22		
	400–500	20.5		
	500–600	11		
Sodium acetate-treated AP fibres	40–120	10.12	0.35	0.020
	120–280	13.19		
	280–400	32.08		
	400–500	20		
	500–600	13		

**Table 5:** Weight percent of C H N S in untreated and sodium acetate-treated AP fibre.

Sample	N%	C%	S%	H%	Sample Weight (mg)
Untreated AP fibre	0.76	43.38	0.51	6.75	14.35
Sodium acetate-treated fibre	0.79	43.47	0.43	ND (not detected)	5.61

**Table 6:** Chemical constituents of untreated and sodium acetate-treated AP fibre.

Chemical Composition	Untreated AP Fibre	Sodium Acetate-Treated
Cellulose, %	45.68	52.59
Hemicellulose, %	41.13	14.51
Lignin, %	24.49	19.4
Pectin, %	12.19	2.92
Moisture, %	13.91	12.7
Wax, %	0.36	0.93
Ash, %	3.13	5.89
Density, g/cc	1.09	0.59

**Table 7:** Mechanical analysis of untreated and sodium acetate-treated AP fibre.

Fibre Sample	Young’s Modulus (GPa)	Elongation at Break (%)	Tensile Strength (MPa)	Standard Deviation (%)
Untreated AP fibre	29.30	6.2	181.694	1.7
Sodium acetate-treated fibre	19.227	2.9	557.58	0.9

## Data Availability

Available on Request.
